# Prevalence of metabolic syndrome and pre-metabolic syndrome in health professionals: LATINMETS Brazil study

**DOI:** 10.1186/s13098-015-0003-x

**Published:** 2015-02-11

**Authors:** Fernanda de Carvalho Vidigal, Andréia Queiroz Ribeiro, Nancy Babio, Jordi Salas-Salvadó, Josefina Bressan

**Affiliations:** Postgraduate Program in Nutrition Science, Department of Nutrition and Health, Universidade Federal de Viçosa, Viçosa, Brazil; Human Nutrition Unit, Department of Biochemistry and Biotechnology, University Hospital Sant Joan de Reus, IISPV, Faculty of Medicine and Health Sciences, Rovira i Virgili University, Tarragona, Spain; CIBERobn Physiopathology of Obesity and Nutrition, Institute of Health Carlos III, Madrid, Spain

**Keywords:** Metabolic syndrome, Prevalence, Health personnel

## Abstract

**Background:**

The metabolic syndrome (MS) is characterized by several cardiovascular risk factors and is associated with an increased incidence of diabetes, cardiovascular events and mortality. The prevalence of MS is increasing in epidemic proportions worldwide. The present study aimed to investigate the prevalence of MS and its components in health professionals in the municipality of Viçosa, Brazil.

**Methods:**

Cross-sectional observational study in the frame of the LATIN America METabolic Syndrome (LATINMETS) multicenter study. The study sample consisted of 226 healthcare personnel (20–59 years). Weight, height, waist circumference and hip circumference were determined. The following anthropometric indices were calculated: body mass index (BMI), waist/hip ratio, waist/height ratio, body adiposity index (BAI) and conicity index. Body composition was assessed by tetrapolar bioelectrical impedance. The lipid profile, fasting glucose, insulin, uric acid, high-sensitive C-reactive protein (hs-CRP) and complement C3 were measured in fasting conditions. Insulin resistance was assessed by the Homeostasis Model Assessment Index of Insulin Resistance (HOMA-IR).

**Results:**

Of the 226 healthcare individuals included in the study, 74.3% were female, 77.0% graduated and 23.0% students of the last two years of courses in health area, with a median age of 27 years. The overall prevalence of MS was 4.5%, and increased with age (20 to 29 years: 1.3%; 30 to 39 years: 5.6%; ≥ 40 years: 26.3%) (P < 0.01). The presence of pre-MS and MS was associated with several measures of adiposity, total cholesterol/HDL-c and LDL-c/HDL-c ratios and serum complement C3 concentrations.

**Conclusions:**

The LATINMETS Brazil study reported an association between MS prevalence and age, especially in those over 40 years. The presence of MS is associated with an increased prevalence of several cardiovascular risk factors.

## Background

Metabolic syndrome (MS) is a set of risk factors for cardiovascular disease, characterized by the presence of abdominal obesity, high fasting glucose, atherogenic dyslipidemia and high blood pressure [1-3]. MS is related to other comorbidities including, prothrombotic state, proinflammatory state, non-alcoholic steatohepatitis and reproductive disorders [4]. MS is also associated with increased risk of type 2 diabetes, cardiovascular disease and mortality from all causes [5]. Furthermore, there is evidence that MS is a simple and effective clinical tool to identify individuals predisposed to high risk of cardiovascular disease and type 2 diabetes. Similarly, components of MS are independently associated with cardiovascular disease and type 2 diabetes, becoming targets of therapeutic changes in lifestyle, medications and surgery [6-8].

Accordingly, the prevalence of MS is increasing in epidemic proportions in both developed countries and developing countries [4]. The worldwide prevalence of MS in the adult population is estimated between 20% and 25% [9]. According to data from the National Health and Nutrition Examination Survey (NHANES) 2009–2010, about one-fifth of the adult population of the United States had high cardiometabolic risk, with the prevalence of MS (adjusted for age) being estimated at 22.9% [8]. Vidigal et al. [10], in a recent systematic review, identified MS prevalence ranging between 4.9% and 65.3% in the adult Brazilian population, including urban, rural and indigenous populations. Health professionals are an important population subgroup, since they are committed to health promotion and prevention, or treatment of diseases, which affect not only their own health but also communities, families and individuals with which they work [11]. The aim of the present study was to assess the prevalence of MS and its components in health professionals in the municipality of Viçosa, Minas Gerais state, Brazil and its association with anthropometric and biochemical indicators.

## Methods

### Study design

This is a cross-sectional observational study conducted in Viçosa, Brazil that integrates the multicentric LATIN America METabolic Syndrome (LATINMETS) study, coordinated by *Universitat Rovira i Virgili* in Reus, Spain, with the same criteria to define the study population and the diagnosis of MS in five Latin American countries (Argentina, Brazil, Colombia, Mexico and Paraguay) [1,11].

The project was approved by the Ethics Committee on Human Research (Of. Ref No 005/2011) of the *Universidade Federal de Viçosa*, in accordance with the principles of the Declaration of Helsinki. Participants were informed about the research objectives and the methodology to be used and signed the Informed Consent Form.

### Target population and the study sample

The population of the Brazil LATINMETS study consisted of health professionals between 20 and 59 years of age. We conducted a survey of 976 healthcare professionals in Viçosa. The calculated sample size was 223 individuals, considering a confidence level of 95%, sampling error of 5% and expected MS prevalence of 25%. Health professionals were invited to participate in the study via phone calls, disclosure on websites, social networks, local radio and pamphlets.

As criteria for inclusion we considered health professional (doctors, nurses, nutritionists, physical trainers, physiotherapists, dentists, pharmacists, biochemists and psychologists), who worked in health facilities and or in a higher education institution, and students in their last two years of courses in the health area. Women who were pregnant or breastfeeding, individuals using steroids or antibiotics at the time of inclusion in the study, individuals who had an illness that required hospitalization at the time of the study, individuals with cancer or who had cancer in the past three years, and individuals who presented difficulties with weighing, measuring, measuring blood pressure or performing blood collection or presented an inflammatory state were excluded. Individuals who had serum high-sensitive C-reactive protein (hs-CRP) above 10 mg/L were also excluded, for being suggestive of inflammation and/or infection in activity [12,13].

### Data collection

Data collection was performed at the Laboratory of Energy Metabolism and Body Composition (LAMECC) and in the Clinical Analyzes Laboratory (LAC), Department of Nutrition and Health at the *Universidade Federal de Viçosa*, from January 2012 to July 2013.

### Sociodemographic assessment and physical activity

Information regarding the clinical history assessing socioeconomic status, personal or family history of diseases, smoking and medication use was recorded. The physical activity was assessed using the long-form International Physical Activity Questionnaire (IPAQ) [14].

### Anthropometric evaluation and body composition

Weight and height were measured according to the techniques recommended by the World Health Organization [15]. Waist circumference (WC) was measured with the aid of flexible, inelastic tape measure at the midpoint between the last rib and the iliac crest [15-18]. Hip circumference (HC) was measured in the area of highest protuberance in the gluteal region with a flexible, inelastic tape measure. Based on the anthropometric measurements, the following anthropometric indices were calculated: body mass index (BMI) that is reached by dividing the weight (kg) by the height (m) squared [15,17]; waist/hip ratio (WHpR) obtained by the ratio between the WC (cm) and the HC (cm) [15]; waist/height ratio (WHtR) obtained by the ratio between the WC (cm) and the height (cm) [19]; conicity index (COI) calculated by the following equation: $WC(m)/0.109\sqrt{weight\ (kg)/ height\ (m)}$ [20] and body adiposity index (BAI) calculated by the equation: $\left[\mathrm{H}\mathrm{C}\left(\mathrm{m}\right)/ height(m)\sqrt{height(m)}\right]-18$ [21].

Body composition was assessed by bioelectrical impedance analysis (BIA), using the Biodynamics Model 310® apparatus, using standardized conditions of measurement [22].

### Blood pressure measurement

Blood pressure was measured using an automatic sphygmomanometer (Omron HEM-742INT), according to the protocol recommended by the European Society of Hypertension and the European Society of Cardiology [23].

### Biochemical determinations

Blood samples were extracted after 12 hours of fast, through intravenous puncture in the median antecubital vein using a vacuum system, with subsequent centrifugation (2,500 rpm, 4°C, 10 min). The samples properly separated and identified were stored at −80°C. Fasting plasma glucose was determined by the glucose oxidase method; uric acid, total cholesterol, HDL-cholesterol (HDL-c) and triglycerides by the enzymatic colorimetric method. LDL-cholesterol (LDL-c) was calculated using the Friedewald formula [24]. Insulin was measured by the electrochemiluminescence method. Plasma hs-CRP and the complement C3 concentrations were measured by nephelometry.

The insulin resistance was assessed by HOMA-IR (Homeostasis Model Assessment - Insulin Resistance) index, calculated by the following equation: [*insulin*(*µU*/*mL*) × *fasting plasma glucose*(*mmol*/*L*)]/22, 5 [25].

### Definition of metabolic syndrome

MS has been defined as the presence of three or more of the five risk factors established by the International Diabetes Federation (IDF) and the American Heart Association/National Heart, Lung, and Blood Institute (AHA/NHLBI) [1]: 1) abdominal obesity (WC ≥ 90 cm in men and ≥ 80 cm in women); 2) hypertriglyceridemia (triglycerides ≥ 150 mg/dL or use of medications to lower triglycerides); 3) low HDL-c (<40 mg/dL in men and <50 mg/dL in women or use of medication to increase HDL-c); 4) high blood pressure (systolic blood pressure ≥130 mmHg and or diastolic blood pressure ≥85 mmHg or use of antihypertensive medication); 5) high fasting plasma glucose ≥100 mg/dL or use of hypoglycemic medication).

### Definition of pre-metabolic syndrome (pre-MS)

Pre-metabolic syndrome (pre-MS) was defined as having no less than two components of MS but did not meet the criteria for the diagnosis of MS [26].

### Statistical analyzes

MS was coded as a dichotomous variable (presence or absence) as well as the number of MS components (discrete continuous variable). In the descriptive statistics the quantitative variables with normal distribution were expressed as mean and standard deviation, and the nonparametric variables as median and interquartile range, according to the Kolmogorov-Smirnov test. Qualitative variables were presented as frequency distribution. We used the chi-square (*χ*^2^) of Pearson tests to compare proportions. For comparison of means the Student *t*-test and analysis of variance (ANOVA) with *post hoc* Tukey test for normally distributed variables were used. Nonparametric variables were analyzed using the Mann–Whitney and Kruskal-Wallis with *post hoc* Dunn's test. Odds ratios (ORs) were estimated to assess the association between the variables studied and the MS and their confidence intervals (CI) of 95% using simple logistic regression analysis. The significance level (α) adopted for all hypothesis tests was 5%. Statistical analysis was performed with SPSS for Windows (version 17.0, SPSS Inc, Chicago, IL).

## Results

238 health professionals aged 20 to 59 years were evaluated, of which 226 concluded the study, constituting the final sample. Of the 226 health professionals included in the study, 74.3% were female, 77.0% graduated and 23.0% students in the last two years of health courses, with an average age of 27 years. According to their areas of expertise, 51.8% were in the nutrition area (64.3% female) and 25.7% in physical education (58.6% men). Regarding socioeconomic status, 56.1% were classified as class B, according to the criteria of the Brazilian Association of Research Companies (ABEP) [27]. The majority (86.1%) had active standard of physical activity and were non-smokers (95.2%). As to medications, 3.1% used antihypertensives; 1.3% hypolipidemics; 0.4% oral hypoglycaemics and/or insulin, with no differences between the sexes, and 39.3% of the women were using contraceptives. Systolic blood pressure, diastolic blood pressure, BMI, WC, HC, WHpR, WHtR, lean mass, fasting glucose and uric acid were higher in men than in women, while BAI, body fat percentage, fat mass, HDL-c, hs-CRP and complement C3 were higher in women (P <0.05) (data not shown).

According to nutritional status, 4.9% (n = 11) of participants were classified as underweight, 73.9% (n = 167) normal weight, 17.7% (n = 40) were overweight and 3.5% (n = 8) obese, according to the World Health Organization classification [28].

Table 1 presents the prevalence of MS and its components. The prevalence of MS was 4.5% (95% CI: 1.7 to 7.2) among health professionals, with no significant difference between sexes. High blood pressure (24.1%), abdominal obesity (20.7%) and low HDL-c (19.0%) were the most prevalent components of MS among men, while abdominal obesity (26.2%), low HDL-c (19.3%) and hypertriglyceridemia (12.0%) were more prevalent in women. The presence of high blood pressure and high fasting plasma glucose MS components was higher among men (P <0.01).

**Table 1:** **Metabolic syndrome and its components in health professionals**

	***Total (n = 226)***	***Men (n = 58)***	***Women (n = 168)***	***P***
*Metabolic syndrome, % (CI 95%)*	4.5 (1.7-7.2)	8.6 (1.2-16.1)	3.0 (0.4-5.6)	0.075
*Metabolic syndrome components, % (CI 95%)*				
*Abdominal obesity*	24.8 (2.9-19.1)	20.7 (9.9-31.4)	26.2 (19.5-32.9)	0.403
*Hypertriglyceridemia*	11.6 (7.4-15.8)	10.3 (2.3-18.4)	12.0 (7.0-17.1)	0.727
*Low HDL-c*	19.2 (14.0-24.4)	19.0 (8.6-29.4)	19.3 (13.2-25.3)	0.959
*High blood pressure*	9.3 (5.5-13.2)	24.1 (12.8-35.5)	4.2 (1.1-7.3)	<0.01
*High fasting plasma glucose*	7.1 (3.7-10.5)	15.5 (5.9-25.1)	4.2 (1.1-7.3)	< 0.01

*Abbreviations:* *CI* confidence interval, *HDL-c* HDL-cholesterol.

Chi-squared Test (*χ* ^*2*^).

Overall prevalence, stratified by sex. Viçosa, Minas Gerais, 2014.

The prevalence of MS was significantly higher in the oldest categories, as well as the prevalence of abdominal obesity and high fasting plasma glucose components (Table 2).

**Table 2:** **Prevalence of metabolic syndrome and its components in health professionals, according to age group**

	***20 to 29 years (n = 151)***	***30 to 39 years (n = 55)***	***≥40 years (n = 20)***	***P***
*Metabolic syndrome, % (CI 95%)*	1.3 (−0.5-3.2)	5.6 (−0.8-11.9)	26.3 (4.5-48.1)	<0.01
*Metabolic syndrome components, % (CI 95%)*				
*Abdominal obesity*	15.2 (9.4-21.0)	34.5 (21.6-47.5)	70.0 (48.0-92.0)	<0.01
*Hypertriglyceridemia*	9.9 (5.1-14.8)	13.0 (3.7-22.2)	21.1 (0.9-41.2)	0.339
*Low HDL-c*	15.9 (10.0-21.8)	22.2 (10.8-33.7)	36.8 (13.0-60.7)	0.074
*High blood pressure*	7.3 (3.1-11.5)	11.1 (2.5-19.8)	20.0 (0.8-39.2)	0.162
*High fasting plasma glucose*	5.3 (1.7-8.9)	9.3 (1.3-17.2)	15.8 (−2.3-33.9)	<0.01

*Abbreviations:* *HDL-c* HDL-cholesterol.

Chi-squared Test (*χ* ^*2*^).

Viçosa, Minas Gerais, 2014.

Table 3 describes the characteristics of health professionals according to the number of MS components as a function of the variables studied. According to gender, 20.7% of men and 11.4% of the women presented two or more components. The presence of three or more components of metabolic syndrome was more common in subjects over 40 years of age (26.3%). Analyzing the education level, 15.7% of graduates and 7.6% of students presented two or more components. According to the area of expertise, graduates, and or nutrition (13.0%) and physical education students (10.3%) had two or more components. Considering the socioeconomic level, the frequency of three or more components was similar between the highest (13.3%) and the lowest class (11.1%). The presence of three or more components was higher among the non-active (12.9%) compared to active individuals (3.1%). Analyzing the nutritional status, 20.8% of overweight individuals had three or more MS components, while no eutrophic subjects exhibited more than two components. It is noteworthy that no health professional presented all five MS components.

**Table 3:** **Number of metabolic syndrome components according to the health professional characteristics**

***Variables***	***n***	***Number of components (%)***		
		***0-1***	***2***	***≥3***
*Gender*				
Men	58	79.3	12.1	8.6
Women	166	88.6	8.4	3.0
*Age (years)*				
20 to 29	151	92.7	6.0	1.3
30 to 39	54	77.8	16.7	5.5
≥40	19	57.9	15.8	26.3
*Educational Level*				
Graduates	172	84.3	11.0	4.7
Students	52	92.4	3.8	3.8
*Area*				
Nutrition	115	87.0	10.4	2.6
Physical Education	58	89.7	8.6	1.7
Others	51	80.4	7.8	11.8
Socioeconomic level				
Class A	30	83.4	3.3	13.3
Class B	119	84.9	11.8	3.3
Class C	53	86.8	11.3	1.9
Class D	9	88.9	0.0	11.1
*Life Style*				
Non-active	31	74.2	12.9	12.9
Active	191	88.0	8.9	3.1
*Smoking*				
Non-smoker	198	85.9	10.1	4.0
Smoker	7	85.7	0.0	14.3
Ex-smoker	2	100.0	0.0	0.0
*BMI (kg/m* ^*2*^ *)*				
<25	176	94.3	5.7	0.0
≥25	48	56.3	22.9	20.8

*Abbreviations:* *BMI* body mass index.

Viçosa, Minas Gerais, 2014.

Diastolic blood pressure, BMI and HC increased with the number of MS components. Health professionals with two or more components showed higher values of age, weight, WC, WHpR, COI, WHtR, body fat percentage, fat mass, triglycerides, and HOMA-IR and lower HDL-c compared with those who had one or no component. Health professionals with MS had higher levels of systolic blood pressure, insulin and complement C3 compared with subjects with one or no component. Those with MS had higher BAI values and higher uric acid concentrations compared to individuals without MS. Height, total cholesterol, fasting glucose and hs-CRP did not differ between groups (Tables 4 and 5).

**Table 4:** **Number of metabolic syndrome components according to age, blood pressure and adiposity indicators**

	***Number of components***			***P***
	***0-1***	***2***	***≥3***	
	***(n = 193)***	***(n = 21)***	***(n = 10)***	
Age (years)*	26 (24–30)^a^	30 (26.0-35.5)^b^	37.5 (28.3-46.0)^b^	<0.01
*Blood pressure*				
Systolic (mmHg) *	105.3 (100.1-114.4)^a^	116 (102.5-125.0)^ab^	125.5 (119.7-132.6)^b^	<0.01
Diastolic (mmHg)^†^	66.1 ± 6.8^a^	71.7 ± 9.0^b^	79.3 ± 8.4^c^	<0.01
*Anthropometric indicators*				
Weight (kg)*	59.3 (53.9-68.1)^a^	68.9 (60.8-82.3)^b^	84.7 (73.3-90.2)^b^	<0.01
Height (cm)^†^	1.67 ± 0.08^a^	1.69 ± 0.08^a^	1.67 ± 0.09^a^	0.669
BMI (kg/m^2^)^†^	22.0 ± 2.7^a^	25.0 ± 3.6^b^	30.6 ± 4.9^c^	<0.01
WC (cm)*	76 (71–82)^a^	85 (80.0-92.7)^b^	103.8 (89.3-107.4)^b^	<0.01
HC (cm)^†^	97.9 ± 5.7^a^	101.7 ± 6.8^b^	108.2 ± 11.4^c^	<0.01
WHpR*	0.78 (0.74-0.83)^a^	0.83 (0.79-0.90)^b^	0.94 (0.90-1.01)^b^	<0.01
BAI^†^	27.4 ± 3.5^a^	28.5 ± 4.3^a^	32.2 ± 4.8^b^	<0.01
COI*	1.16 (1.12-1.20)^a^	1.21 (1.17-1.28)^b^	1.33 (1.26-1.37)^b^	<0.01
WHtR*	0.45 (0.43-0.49)^a^	0.51 (0.47-0.54)^b^	0.62 (0.55-0.68)^b^	<0.01
*Body composition*				
Body fat (%)^†^	21.9 ± 6.1^a^	26.1 ± 6.6^b^	28.8 ± 8.5^b^	<0.01
Fat mass (kg)*	12.9 (10.4-15.8)^a^	17 (15.3-20.3)^b^	24 (16.7-29.9)^b^	<0.01
Lean mass (kg)*	45.2 (41.6-52.7)^a^	50.2 (44.9-64.0)^ab^	58.7 (49.0-68.8)^b^	<0.01

*Abbreviations:* *BMI* body mass index, *WC* waist circumference, *HC* hip circumference, *WHpR* waist/hip ratio, *BAI* body adiposity index, *COI* conicity index, *WHtR* waist/height ratio.

*Nonparametric variables.

^†^parametric variables.

ANOVA with *post hoc* Tukey test for parametric variables. Kruskal-Wallis test with *post hoc* Dunn’s test for nonparametric variables. Comparison between columns. Different letters indicate statistical difference between the values presented.

Viçosa, Minas Gerais, 2014.

**Table 5:** **Number of metabolic syndrome components according to biochemical parameters**

	***Number of components***			***P***
	***0-1***	***2***	***≥3***	
	***(n = 193)***	***(n = 21)***	***(n = 10)***	
*Biochemical parameters*				
Total cholesterol (mg/dL)^†^	183.5 ± 35.9^a^	190.1 ± 40.1^a^	183.9 ± 39.9^a^	0.735
HDL-c (mg/dL)^†^	61.3 ± 14.6^a^	51.2 ± 14.3^b^	39.7 ± 6.9^b^	<0.01
Triglycerides (mg/dL)*	82 (58–105)^a^	115 (79–150)^b^	206.5 (101.5-251.5)^b^	<0.01
Fasting glucose (mg/dL)*	85.5 (80–92)^a^	90 (80.5-101.0)^a^	93.5 (86–102)^a^	0.052
Insuline (μUI/mL)*	6.2 (4.4-8.6)^a^	7.8 (5.8-9.6)^ab^	13.9 (7.6-17.5)^b^	<0.01
HOMA-IR*	1.29 (0.90-1.84)^a^	1.75 (1.20-2.02)^b^	2.85 (1.79-3.98)^b^	<0.01
Uric acid (mg/dL)^†^	3.99 ± 1.14^a^	4.2 ± 0.91^a^	5.4 ± 1.53^b^	<0.01
hs-CRP (mg/L)*	1.00 (0.65-2.00)^a^	1.50 (0.64-6.25)^a^	3.00 (0.72-5.50)^a^	0.231
Complement C3 (mg/dL)^†^	103.7 ± 17.7^a^	113.4 ± 14.6^ab^	127.7 ± 22.7^b^	<0.01

*Abbreviations:* *HDL-c* HDL-cholesterol, *HOMA-IR* Homeostasis Model Assessment - Insulin Resistance, *hs-CRP* high-sensitive C-reactive protein.

*Nonparametric variables.

^†^parametric variables.

ANOVA with *post hoc* Tukey test for parametric variables. Kruskal-Wallis test with *post hoc* Dunn’s test for nonparametric variables. Comparison between columns. Different letters indicate statistical difference between the values presented.

Viçosa, Minas Gerais, 2014.

The pre-MS, i.e., the presence of two MS components, was associated with BMI, body fat percent, total cholesterol/HDL-c and LDL-c/HDL-c ratios and complement C3. For each increase of 1 kg/m^2^ in BMI and 1% in body fat there was an increase of 36% and 12% in the risk of pre-MS. Regarding biochemical indices evaluated, for each increase of 1 unit in total cholesterol/HDL-c and LDL-c/HDL-c ratios, the risk for pre-MS was 2.53 fold. For each increase of 1 mg/dL of complement C3 concentrations, the risk of MS was 1.03 fold higher. MS was positively associated with age, physical inactivity, BMI, BAI, body fat percentage, total cholesterol/HDL-c and LDL-c/HDL-c ratios, uric acid, and complement C3. Individuals aged 40 or older were 14.3 times more likely to have MS compared to those less than 40 years. Non-active health professionals had 4.6 times more risk to have MS than the active professionals. For each 1 kg/m^2^ increase in BMI, 1 unit of BAI and 1% body fat, there was a 75%, 32% and 18% increased risk of MS, respectively. Regarding biochemical indices evaluated, for each increase of 1 unit in total cholesterol/HDL-c and LDL-c/HDL-c ratios, the risk for having MS was 3.38 and 2.49 fold higher, respectively. For each increase of 1 mg/dL in the concentrations of uric acid and complement C3, the risk of having MS was 2.06 and 1.06 times higher, respectively (Table 6).

**Table 6:** **Odds ratio for the association between age, physical activity patterns, adiposity indicators and biochemical parameters, and pre-metabolic syndrome and metabolic syndrome, in health professionals**

**Variables**	**Pre-MS**	**MS**
	**OR (CI 95%)**	**OR (CI 95%)**
Age (≥40 years)	2.76 (0.70-10.80)	14.29 (3.69-55.26)
Physical activity patterns (non-active)	1.72 (0.53-5.55)	4.57 (1.21-17.24)
BMI (kg/m^2^)	1.36 (1.17-1.58)	1.75 (1.35-2.27)
BAI	1.08 (0.96-1.22)	1.32 (1.13-1.56)
Body fat (%)	1.12 (1.04-1.21)	1.18 (1.06-1.32)
Total cholesterol /HDL-c	2.53 (1.54-4.15)	3.38 (1.89-6.05)
LDL-c/HDL-c	2.53 (1.44-4.46)	2.49 (1.28-4.86)
Uric acid (mg/dL)	1.18 (0.79-1.75)	2.06 (1.32-3.22)
Complement C3 (mg/dL)	1.03 (1.00-1.06)	1.06 (1.02-1.09)

*Abbreviations:* *Pre-MS* pre-metabolic syndrome, *MS* metabolic syndrome, *OR* odds ratio, *CI* confidence interval, *BMI* body mass index, *BAI* body adiposity index, *HDL-c* HDL-cholesterol, *LDL-c* LDL-cholesterol.

Viçosa, Minas Gerais, 2014.

## Discussion

The LATINMETS Brazil study reported an overall MS prevalence of 4.5% (95% CI: 1.7 to 7.2) in health care workers, with no significant difference between the sexes, according to the criteria established by the IDF and the AHA/NHLBI. The prevalence of MS reported in this study was lower than previously published, according to different criteria for defining the MS [7,8,10,11,29-31]. The LATINMETS Columbia study identified an MS prevalence of 17.5% among the health professionals evaluated (n = 285), using the same criteria of the present study [11]. NHANES data show a reduction in the prevalence of MS in the United States from 25.5% in 1999–2000 to 22.9% in 2009–2010, according to the criteria established by the IDF and the AHA/NHLBI [8]. In Mexico, the prevalence of MS ranged between 13.6% and 26.6% depending on the diagnostic criteria used [29]. The overall prevalence estimated by the CARMELA study that evaluated MS in seven Latin American countries (Argentina, Chile, Colombia, Ecuador, Mexico, Peru and Venezuela) was 21.0%, according to the criteria of the National Cholesterol Education Program Adult Treatment Panel III (NCEP-ATP III) [7]. Bustos et al. identified MS prevalence of 7.6% in an urban population in Brazil and 10.1% in a semi-rural population of Chile [30], while Silva et al. [31] reported MS prevalence of 15.4% in a rural population in Brazil, according to the criteria of NCEP-ATP III. A recent systematic review estimated the prevalence of MS at 29.6% of the adult Brazilian population, using different criteria for the definition of MS [10].

Although the overall prevalence of MS was low, it was found that it increased with age, presenting as elevated in individuals over 40 years of age (26.3%). This finding was also reported by the LATINMETS Colombia study [11]. Corroborating these findings, all Latin American countries evaluated by the CARMELA study showed an increased prevalence of MS with age [7], as in the studies of the Brazilian population [32,33].

According to socioeconomic status, the prevalence of MS was similar between the highest level (Class A) and the lowest (Class D) of the sample. It is noteworthy that the majority (70.7%) belonged to the highest socioeconomic levels (Classes A and B) and only 4.2% were Class D, with no participant framed in Class E. Buckland et al. [2] found that MS remained associated with low socioeconomic status, after adjustment for confounders. Accordingly, Bustos et al. [30] assessed the prevalence of MS in two countries with different socioeconomic levels, and their results suggest the existence of a cultural, educational and socioeconomic phenomenon that possibly influences the prevalence of diagnosis of MS components based on differences in lifestyle, rather than belonging to a particular place. It is noteworthy that the sample was composed of health professionals and, or, higher education students, thus homogeneous as to the degree of education. Therefore, our results are not comparable when considering the general population.

MS was more frequent among non-active health professionals and was present only in individuals who are overweight. The combination of physical inactivity and obesity are associated with insulin resistance, which may explain the increased prevalence of MS in these individuals [4,34,35]. The LATINMETS Colombia study found that the prevalence of MS in individuals with excess weight was ten times higher compared to those with a normal BMI [11].

HC and BMI increased with the number of MS components. Health professionals with two or more components presented higher values of age, weight, WHpR, COI, WHtR, body fat percentage, fat mass, triglycerides, and HOMA-IR and lower HDL-c compared with those who had one or no component. It appears that the presence of two MS components is capable of identifying higher values of anthropometric indicators of obesity, atherogenic dyslipidemia, and insulin resistance index. It can be speculated pre-MS [26,36,37] is able to identify anthropometric indicators and metabolic changes, showing that this early stage preceding the MS is also characterized by an increased risk of atherosclerotic complications and insulin resistance.

In the present study, the prevalence of pre-MS was 12.1% and 8.4% in men and women respectively. It is noteworthy that when comparing individuals with pre-MS with those with MS, few parameters were statistically different, a fact that reveals a rather unsatisfactory health profile, in those even without installed MS.

Health professionals with MS had higher insulin, and complement C3 concentrations compared with subjects with one or no component. Subjects with MS showed higher BAI and higher uric acid concentrations compared with those without MS. The BAI, based on measurements of HC and height, has been suggested as an adiposity index better correlated with the body fat percentage than the BMI [21,38]. Data from the study Triglyceride and Cardiovascular Risk in African-Americans (TARA), identified a correlation between the body fat percentage, assessed by Dual Emission X-ray absorptiometry (DXA), and a BAI of 0.849 (P <0.001) [21]. However, Zhang et al. [38] found that the BAI was not better than WC and BMI in predicting body fat percentage, metabolic abnormalities and subclinical atherosclerosis in Chinese populations. Hyperuricemia, even in asymptomatic clinical manifestations, is a cardiometabolic risk factor in adults [37]. In agreement with the present study, Desai et al. [39] demonstrated that concentrations of uric acid increased linearly with the increasing number of MS components in Brazilian men (46 ± 7 years old). In addition, uric acid has been found to be a reliable indicator of pre-MS in obese adolescents (10 to 15.9 years old) [37]. Corroborating these data, studies in Asian populations (China, South Korea, Thailand and Japan) also found an association between metabolic syndrome and uric acid concentrations [40-43]. Phillips et al. [44] reported a three-fold higher risk of having MS and its phenotypes, including abdominal obesity, insulin resistance and low concentrations of HDL-c in subjects with higher concentrations of complement C3 (> median), an acute phase protein with an important function in the innate immune system. Onat et al. [45] demonstrated that the complement C3 were positively associated with WC, triglycerides and hs-CRP concentrations, and inversely with smoking status. Moreover, the concentrations of complement C3 were able to predict the presence of MS.

In the present study, the pre-MS and the MS were associated with BMI, percent total body fat, total cholesterol/HDL-c and LDL-c/HDL-c ratios and complement C3 concentrations. In addition to these variables, the MS was positively associated with age, physical inactivity, BAI and uric acid. Buckland et al. found that, after adjustment for confounders, the MS was positively associated with the male gender, age, BMI, physical inactivity and low socioeconomic status, whereas smoking and marital status were not associated with MS [2]. Participants of the Coronary Artery Risk Development in Young Adults (CARDIA) study with higher initial BMI who did not drink alcohol (compared with one to three drinks per day), with higher carbohydrate intake and lower fiber intake had higher MS risk (relative risk: 1.3 to 1.9), while the physical activity had a protective association (relative risk: 0.84, 95% CI: 0.76 to 0.92) [46].

The main limitations of the present study are the characteristics inherent in a cross-sectional study, i.e., the associations between MS and variables cannot be interpreted as causal associations. Furthermore, one should be cautious when interpreting the differences between the sexes due to the small sample of men in our study. Another limitation presented refers to the questionnaire used to assess physical activity. The IPAQ, despite being an internationally validated instrument, is not the best method to estimate physical activity, because it tends to overestimate the number of active individuals [47]. The ideal would be to use pedometers or accelerometers. However, among the available instruments in this study, the IPAQ was the most appropriate.

## Conclusions

The LATINMETS Brazil study reported low overall prevalence of MS among health professionals compared with data from the scientific literature. We observed an increase in the prevalence of MS with age, verifying high prevalence in individuals over 40. MS was associated with changes in several anthropometric and metabolic indicators, and was present only in individuals who are overweight. Pre-MS and MS were associated with measures of adiposity, total cholesterol/HDL-c and LDL-c/HDL-c ratios and complement C3, revealing a rather unsatisfactory health profile even in individuals without MS installed. It is up to government agencies and health professionals to discuss the issue and the implementation of public health policies with the aim of contributing to excess weight reduction and the incentive to practice healthy lifestyle habits, and consequent prevention of this problem in the population.

## Abbreviations

AHA/NHLBI: American Heart Association/National Heart, Lung, and Blood Institute
BAI: Body adiposity index
BMI: Body mass index
CI: Confidence interval
HC: Hip circumference
HDL-c: HDL-cholesterol
HOMA-IR: Homeostasis Model Assessment Index of Insulin Resistance
hs-CRP: High-sensitive C-reactive protein
IDF: International Diabetes Federation
IPAQ: International Physical Activity Questionnaire
LATINMETS: LATIN America METabolic Syndrome
LDL-c: LDL-cholesterol
MS: Metabolic syndrome
pre-MS: Pre-metabolic syndrome
WC: Waist circumference
WHpR: Waist/hip ratio
WHtR: Waist/height ratio

## References

[1] Alberti KGMM, Eckel RH, Grundy SM, Zimmet PZ, Cleeman JI, Donato KA (2009). Harmonizing the metabolic syndrome: A joint interim statement of the International Diabetes Federation Task Force on Epidemiology and Prevention; National Heart, Lung, and Blood Institute; American Heart Association; World Heart Federation; International Atherosclerosis Society; and International Association for the Study of Obesity. Circulation.

[2] Buckland G, Salas-Salvadó J, Roure E, Bulló M (2008). L. S-M. Sociodemographic risk factors associated with metabolic syndrome in a Mediterranean population. Public Health Nutr.

[3] Ogbera AO (2010). Prevalence and gender distribution of the metabolic syndrome. Diabetol Metab Syndr.

[4] Cornier MA, Dabelea D, Hernandez TL, Lindstrom RC, Steig AJ, Stob NR (2008). The metabolic syndrome. Endocr Rev.

[5] Ford ES (2005). Risks for all-cause mortality, cardiovascular disease, and diabetes associated with the metabolic syndrome: a summary of the evidence. Diabetes Care.

[6] McNeill AM, Rosamond WD, Girman CJ, Golden SH, Schmidt MI, East HE (2005). The metabolic syndrome and 11-year risk of incident cardiovascular disease in the atherosclerosis risk in communities study. Diabetes Care.

[7] Escobedo J, Schargrodsky H, Champagne B, Silva H, Boissonnet C, Vinueza R (2009). Prevalence of the metabolic syndrome in Latin America and its association with sub-clinical carotid atherosclerosis: the CARMELA cross sectional study. Cardiovasc Diabetol.

[8] Beltrán-Sánchez H, Harhay MO, Harhay MM, McElligott S (2013). Prevalence and trends of Metabolic Syndrome in the adult US population, 1999–2010. J Am Coll Cardiol.

[9] International Diabetes Federation. The IDF consensus worldwide definition of the metabolic syndrome. 2006. http://www.idf.org/webdata/docs/IDF_Meta_def_final.pdf.

[10] Vidigal FC, Bressan J, Babio N, Salas-Salvadó J (2013). Prevalence of metabolic syndrome in Brazilian adults: a systematic review. BMC Public Health.

[11] González-Zapata LI, Deossa GC, Monsalve-Álvarez J, Díaz-García J, Babio N, Salas-Salvadó J (2013). Metabolic syndrome in healthcare personnel of the university of Antioquia-Colombia. LATINMETS study. Nutr Hosp.

[12] Petersson H, Daryani A, Risérus U (2007). Sagittal abdominal diameter as a marker of inflammation and insulin resistance among immigrant women from the Middle East and native Swedish women: a cross-sectional study. Cardiovasc Diabetol.

[13] Brasil AR, Norton RC, Rossetti MB, Leão E, Mendes RP (2007). C-reactive protein as an indicator of low intensity inflammation in children and adolescents with and without obesity. J Pediatr.

[14] Matsudo S, Araujo T, Matsudo V, Andrade D, Andrade E, Oliveira LC (2001). Questionário internacional de atividade física (IPAQ): estudo de validade e reprodutibilidade no Brasil. Atividade física e saúde.

[15] Organización Mundial de la Salud (1995). El estado físico: uso e interpretación de la antropometría: informe de un Comitê de Expertos de la OMS. OMS, Serie de Informes Técnicos 854.

[16] Sociedade Brasileira de Hipertensão, Sociedade Brasileira de Cardiologia, Sociedade Brasileira de Endocrinologia e Metabologia, Sociedade Brasileira de Diabetes, Sociedade Brasileira de Estudos da Obesidade (2005). I Diretriz brasileira de diagnóstico e tratamento da síndrome metabólica. Arq Bras Cardiol.

[17] World Health Organization (2000). Obesity: preventing and managing the global epidemic. Report of a WHO Consultation. WHO Technical Report Series 894.

[18] Pitanga FJG, Lessa I (2004). Sensibilidade e especificidade do índice de conicidade como discriminador do risco coronariano de adultos em Salvador. Brasil Rev Bras Epidemiol.

[19] Pitanga FJG, Lessa I (2006). Razão cintura-estatura como discriminador do risco coronariano de adultos. Rev Assoc Med Bras.

[20] Valdez R (1991). A simple model-based index of abdominal adiposity. J Clin Epidemiol.

[21] Bergman RN, Stefanovski D, Buchanan TA, Sumner AE, Reynolds JC, Sebring NG (2011). A better index of body adiposity. Obesity.

[22] Rezende FAC, Rosado LEFPL, Ribeiro RCL, Vidigal FC, Vasques ACJ, Bonard IS (2006). Body mass index and waist circumference: association with cardiovascular risk factors. Arq Bras Cardiol.

[23] Mancia G, De Backer G, Dominiczak A, Cifkova R, Fagard R, Germano G (2007). 2007 Guidelines for the Management of Arterial Hypertension: The Task Force for the Management of Arterial Hypertension of the European Society of Hypertension (ESH) and of the European Society of Cardiology (ESC). J Hypertens.

[24] Friedewald WT, Levy RI, Fredrickson DS (1972). Estimation of the concentration of low-density lipoprotein cholesterol in plasma, without use of the preparative ultracentrifuge. Clin Chem.

[25] Matthews DR, Hosker JP, Rudenski AS, Naylor BA, Treacher DF, Turner RC (1985). Homeostasis model assessment: insulin resistance and beta-cell function from fasting plasma glucose and insulin concentrations in man. Diabetologia.

[26] Yin Q, Chen X, Li L, Zhou R, Huang J, Yang D (2013). Apolipoprotein B/apolipoprotein A1 ratio is a good predictive marker of metabolic syndrome and pre-metabolic syndrome in Chinese adolescent women with polycystic ovary syndrome. J Obstet Gynaecol Res.

[27] Associação Brasileira de Empresas de Pesquisa (ABEP). Critério de Classificação Econômica Brasil. 2012. http://www.abep.org/criterioBrasil.aspx.

[28] World Health Organization (WHO) (2002). Diet, Nutrition and the Prevention of Chronic Diseases: Report of a Joint WHO/FAO Expert Consultation. WHO Technical Report Series.

[29] Aguilar-Salinas CA, Rojas R, Gómez-Pérez FJ, Valles V, Ríos-Torres JM, Franco A (2004). High prevalence of metabolic syndrome in Mexico. Arch Med Res.

[30] Bustos P, da Silva AAM, Amigo H, Bettiol H, Barbieri MA (2007). Metabolic syndrome in young adults from two socioeconomic Latin American settings. Nutr Metab Cardiovasc Dis.

[31] Silva KF, Prata A, Cunha DF (2011). Frequency of metabolic syndrome and the food intake patterns in adults living in a rural area of Brazil. Rev Soc Bras Med Trop.

[32] Marquezine GF, Oliveira CM, Pereira AC, Krieger JE, Mill JG (2008). Metabolic syndrome determinants in an urban population from Brazil: Social class and gender-specific interaction. Int J Cardiol.

[33] Salaroli LB, Barbosa GC, Mill JG, Molina MCB (2007). Prevalência de síndrome metabólica em estudo de base populacional, Vitória, ES - Brasil. Arq Bras Endocrinol Metabol.

[34] Alley DE, Chang VW (2010). Metabolic syndrome and weight gain in adulthood. J Gerontol A Biol Sci Med Sci.

[35] Thabit H, Burns N, Shah S, Brema I, Crowley V, Finnegan F (2013). Prevalence and predictors of diabetes and cardiometabolic risk among construction workers in Ireland: the Construction Workers Health Trust screening study. Diab Vasc Dis Res.

[36] Dimitrijevic-Sreckovic V, Colak E, Djordjevic P, Gostiljac D, Sreckovic B, Popovic S (2007). Prothrombogenic factors and reduced antioxidative defense in children and adolescents with pre-metabolic and metabolic syndrome. Clin Chem Lab Med.

[37] Denzer C, Muche R, Mayer H, Heinze E, Debatin KMMW (2003). Serum uric acid levels in obese children and adolescents: linkage to testosterone levels and pre-metabolic syndrome. J Pediatr Endocrinol Metab.

[38] Zhang ZQ, Liu YH, Xu Y, Dai XW, Ling WH, Su YX (2014). The validity of the body adiposity index in predicting percentage body fat and cardiovascular risk factors among Chinese. Clin Endocrinol.

[39] Desai MY, Santos RD, Dalal D, Carvalho JA, Martin DR, Flynn JA (2005). Relation of serum uric acid with metabolic risk factors in asymptomatic middle-aged Brazilian men. Am J Cardiol.

[40] Dai X, Yuan J, Yao P, Yang B, Gui L, Zhang X (2013). Association between serum uric acid and the metabolic syndrome among a middle- and old-age Chinese population. Eur J Epidemiol.

[41] Lee JM, Kim HC, Cho HM, Oh SM, Choi DP, Suh I (2012). Association between serum uric acid level and metabolic syndrome. J Prev Med Public Health.

[42] Jaipakdee J, Jiamjarasrangsri W, Lohsoonthorn V, Lertmaharit S (2013). Prevalence of metabolic syndrome and its association with serum uric acid levels in Bangkok Thailand. Southeast Asian J Trop Med Public Health.

[43] Oda E (2014). Serum uric acid is an independent predictor of metabolic syndrome in a Japanese health screening population. Heart Vessels.

[44] Phillips CM, Kesse-Guyot E, Ahluwalia N, McManus R, Hercberg S, Lairon D (2012). Dietary fat, abdominal obesity and smoking modulate the relationship between plasma complement component 3 concentrations and metabolic syndrome risk. Atherosclerosis.

[45] Onat A, Hergenç G, Can G, Kaya Z, Yüksel H (2010). Serum complement C3: a determinant of cardiometabolic risk, additive to the metabolic syndrome, in middle-aged population. Metabolism.

[46] Carnethon MR, Loria CM, Hill JO, Sidney S, Savage PJ, Liu K (2004). Risk factors for the metabolic syndrome: the Coronary Artery Risk Development in Young Adults (CARDIA) study, 1985–2001. Diabetes Care.

[47] Rzewnicki R, Vanden Auweele Y, De Bourdeaudhuij I (2003). Addressing overreporting on the International Physical Activity Questionnaire (IPAQ) telephone survey with a population sample. Public Health Nutr.

